# Supplementary material for: Structural basis of ligand selectivity and disease mutations in cysteinyl leukotriene receptors
Source: Nat Commun. 2019 Dec 6;10:5573. doi: 10.1038/s41467-019-13348-2 (PMC6897958; doi:10.1038/s41467-019-13348-2)
Supplement: Supplementary file 1 — Supplementary Information [file 41467_2019_13348_MOESM1_ESM.pdf]

SUPPLEMENTARY INFORMATION

Structural Basis of Ligand Selectivity and Disease Mutations in Cysteinyl Leukotriene Receptors

Gusach, Luginina *et al*.

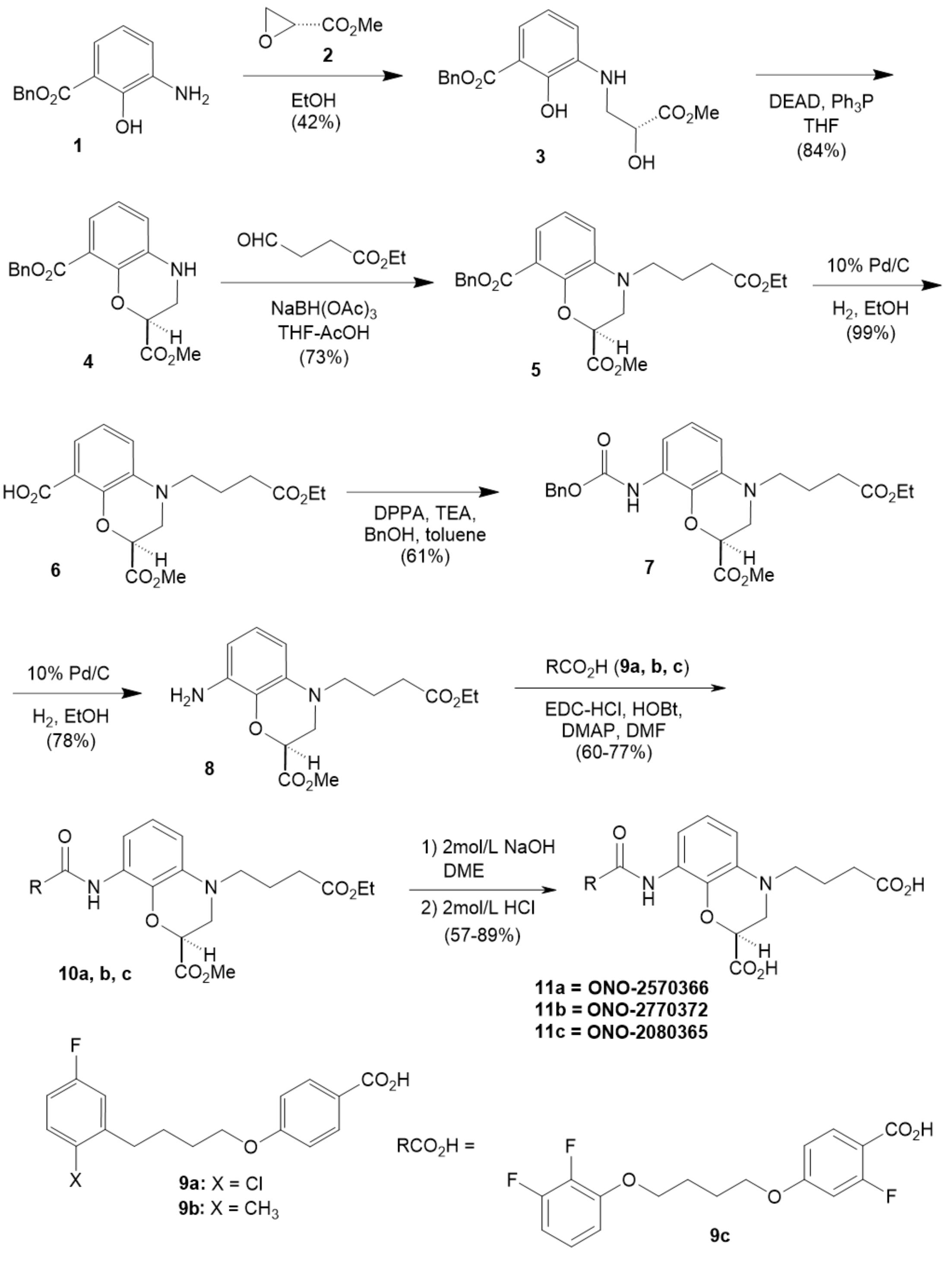

Supplementary Figure 1. Synthesis scheme for ONO-2570366 (11a), ONO-2770372 (11b), and ONO-2080365 (11c).

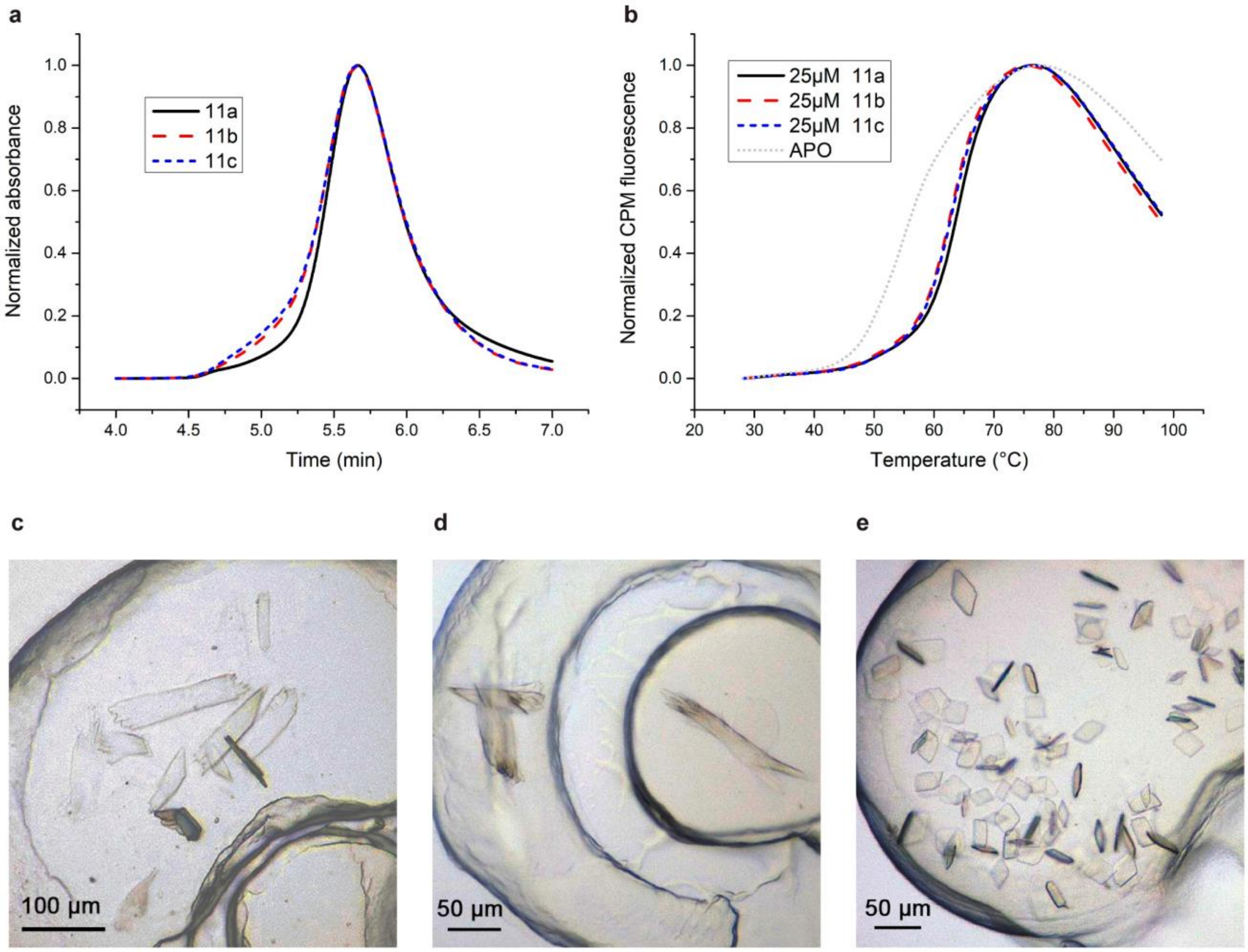

Supplementary Figure 2. Characterization and crystallization of CysLT$_2$R. (a) Analytical size exclusion chromatography analysis of purified CysLT$_2$R in complex with cpds 11a, 11b, and 11c, showing mostly monomeric protein preparations. (**b**) Thermal shift assay using CPM fluorescence. Ligands increase thermal stability of CysLT$_2$R by ~10 °C. (**c-e**) Crystals of CysLT$_2$R-11a (c), CysLT$_2$R-11b (d), and CysLT$_2$R-11c (e) grown in lipidic cubic phase.

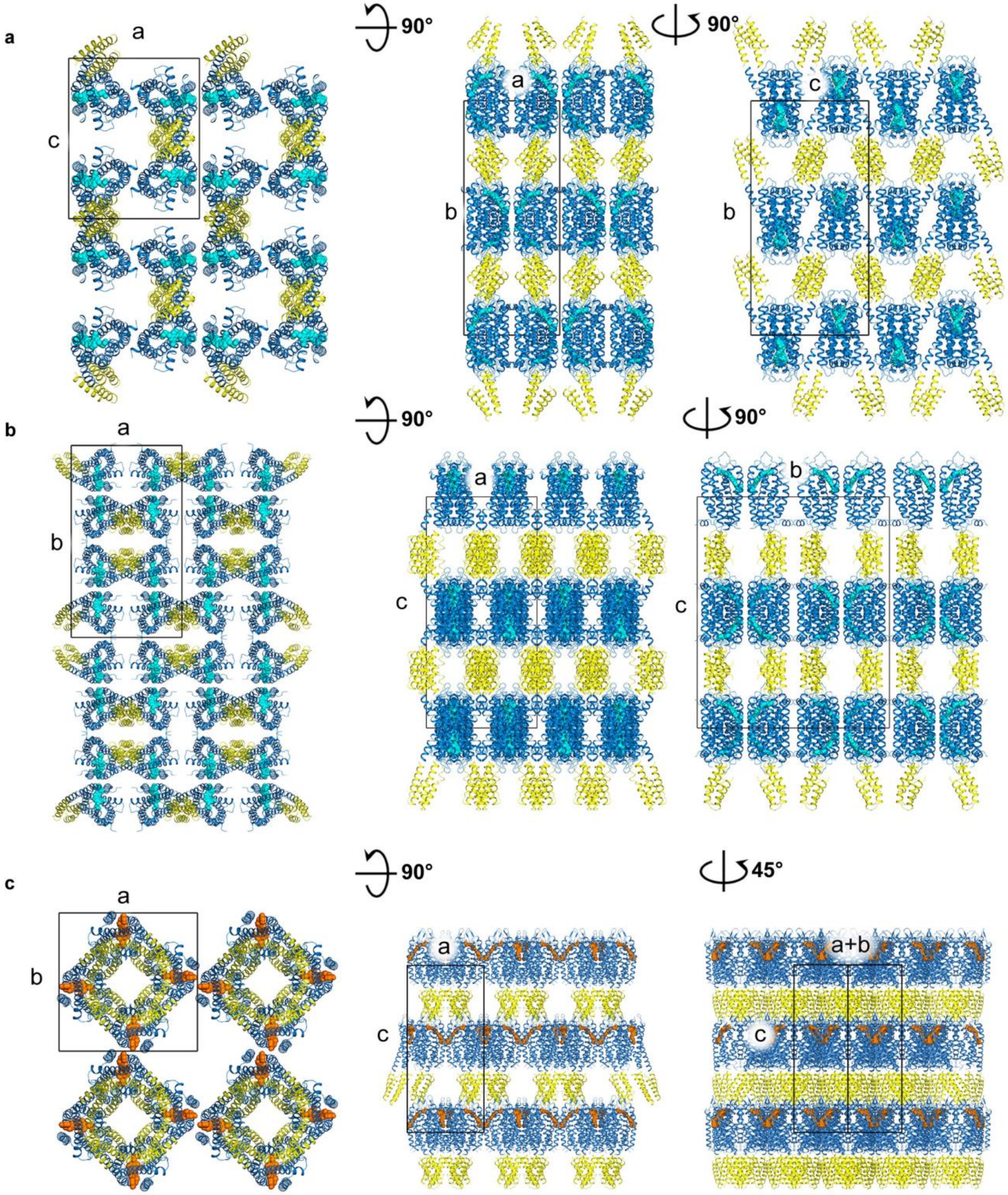

Supplementary Figure 3. Crystal packing. (**a**) CysLT$_2$R-11a in C222$_1$ space group. (**b**) CysLT$_2$R-11a in F222 space group. (**c**) CysLT$_2$R-11c in I4 space group. CysLT$_2$R is shown in blue, BRIL - in yellow, cpd 11a - in cyan, cpd 11c - in orange. For top-views (left panels), only a single crystal layer is shown. Unit cells are shown as boxes with black outlines and corresponding axes are labeled as a, b, and c.

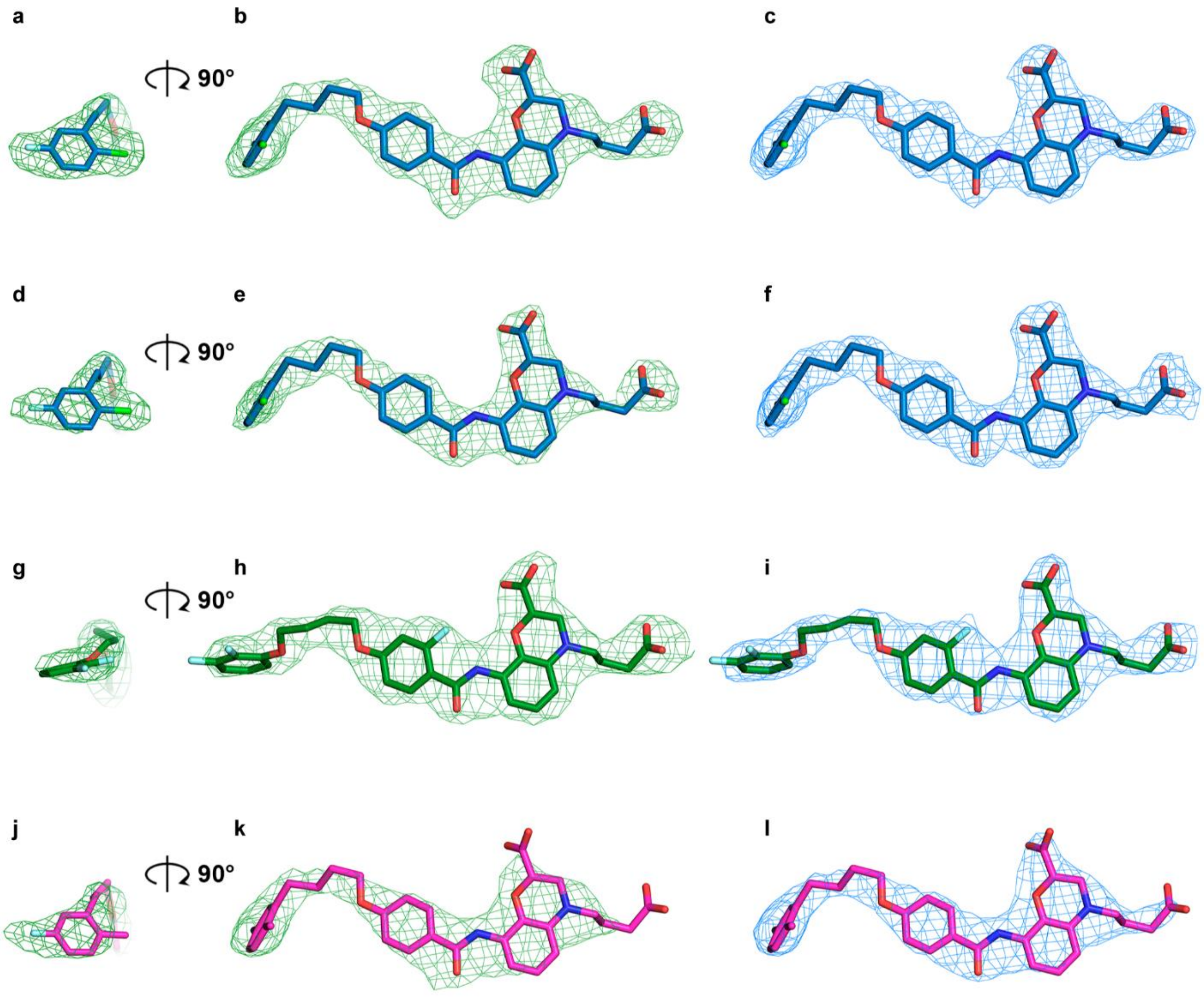

Supplementary Figure 4. Electron density for ligands. (**a-c**) cpd 11a (blue) in $C222_1$ crystal form. (**d-f**) cpd 11a (blue) in F222 crystal form. (**g-i**) cpd 11c (green). (**j-l**), cpd 11b (purple). Simulated annealing ligand omit mFo-DFc density maps (green mesh) contoured at 3 σ level (a, b, d, e, g, h, j, k) and 2mFo-DFc maps (blue mesh) contoured at 1 σ (c, f, i, l) are shown around ligands.

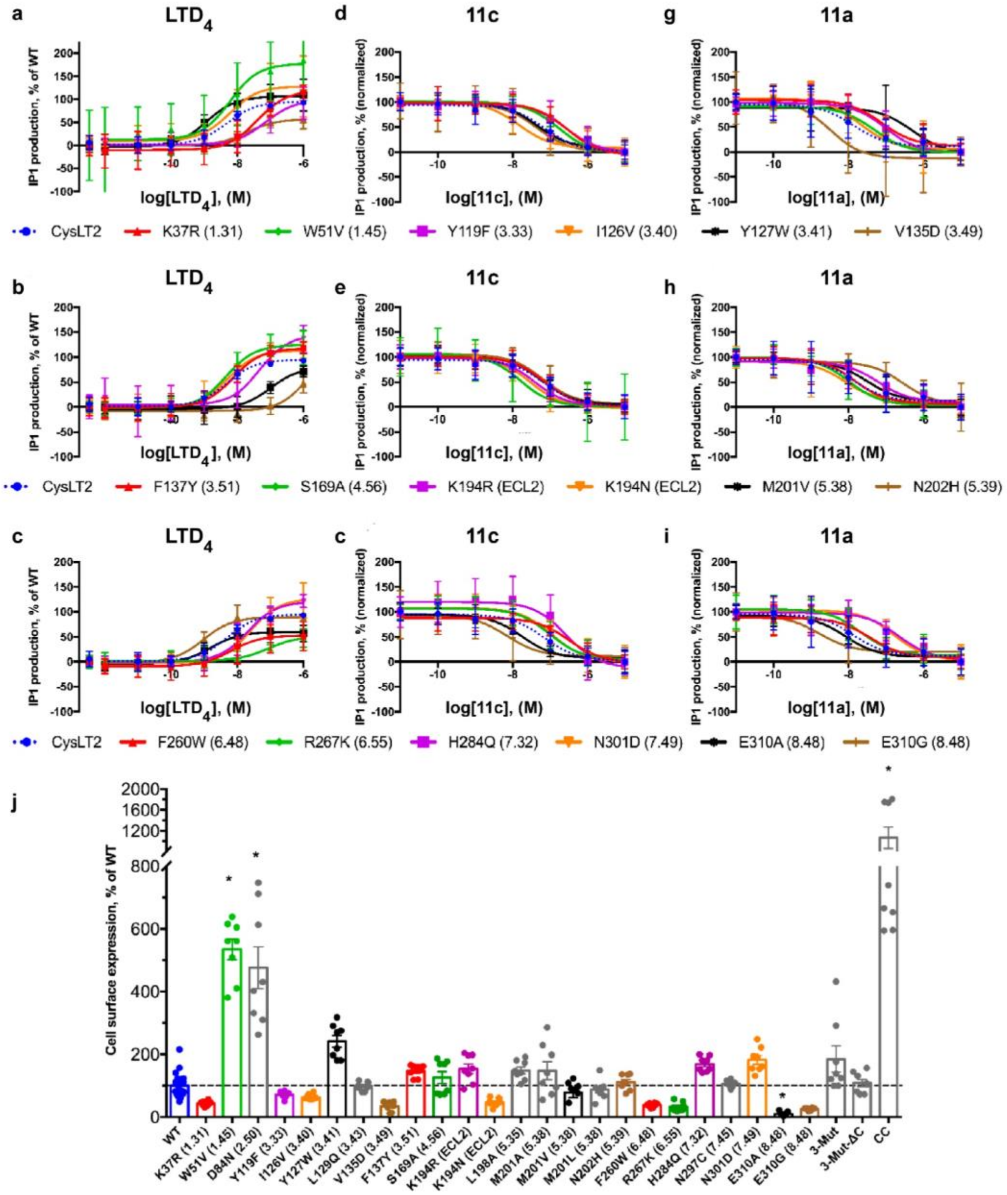

Supplementary Figure 5. Effects of LTD_4 and $CysLT_2R$ antagonists on IP_1 production. (a-c) LTD_4-stimulated IP_1 accumulation at WT $CysLT_2R$ and mutants. **(d-i)** Inhibition of IP_1 production, stimulated with a fixed EC_{80} concentration of LTD_4 (from a-c), by cpd 11c (d-f) and cpd 11a (g-i). Results were normalized for each mutant; non-stimulated cells were set as 0% IP_1 production whereas cells stimulated with LTD_4 alone were set as 100% IP_1 production. Each data point represents mean ± s.d. (the exact numbers of independent experiments, performed in quadruplicate, for each mutant are shown in Table 1). **(j)** Cell surface expression of the HA-tagged WT $CysLT_2R$ and mutants as determined by ELISA. Each data set represents mean ± s.d. (the exact numbers of independent experiments performed in quadruplicates for each mutant are shown in Table 1; * $P < 0.05$ compared to WT $CysLT_2R$ analyzed by the Kruskal-Wallis nonparametric test followed by Dunn's correction). Non-responsive mutants in the IP_1 production assay are shown in gray. "3-Mut" corresponds to WT $CysLT_2R$ with three mutations $W51^{1.45}V$, $D84^{2.50}N$, $F137^{3.51}Y$, used in the crystallized construct (CC). "3-Mut-ΔC" corresponds to WT $CysLT_2R$ with three mutations $W51^{1.45}V$, $D84^{2.50}N$, $F137^{3.51}Y$ and Δ323-346 C-terminal truncation, as used in CC. Source data are provided as a Source Data file.

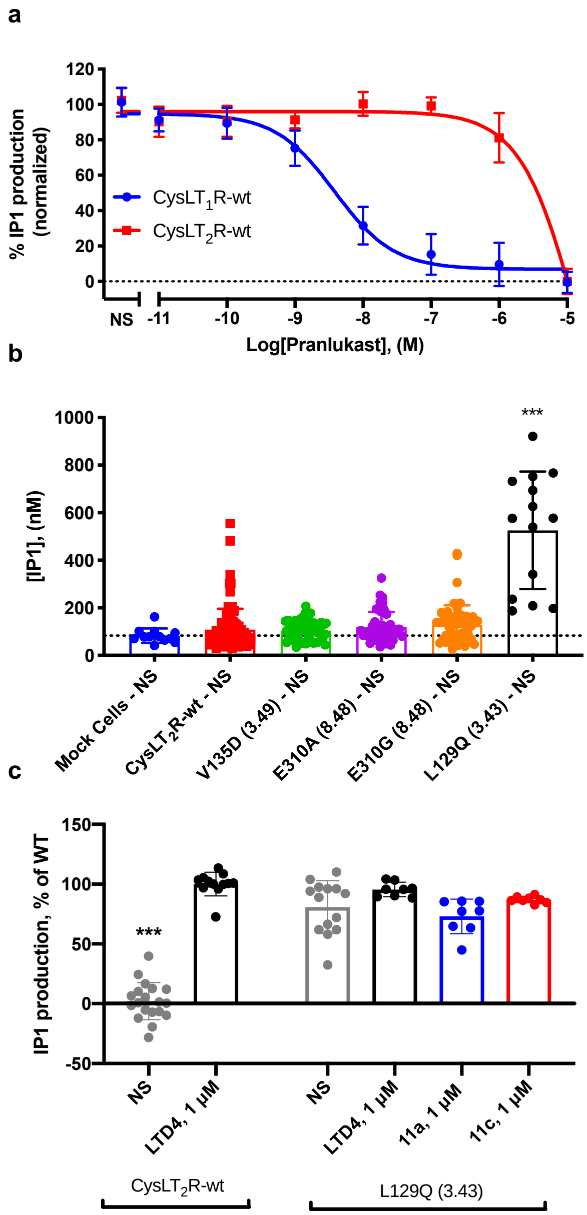

Supplementary Figure 6. Pranlukast selectivity and basal activity of $CysLT_2R$. (a) Inhibition of LTD_4-stimulated IP_1 production by pranlukast in HEK293 cells transiently transfected with the wild-type (WT) $CysLT_1$ and $CysLT_2$ receptors. **(b)** No increased receptor basal activity is observed with the $V135^{3.49}D$, $E310^{8.48}A$, $E310^{38.48}G$ $CysLT_2R$ mutants. In sharp contrast, $L129^{3.43}Q$ is constitutively active (*** $P < 0.001$ compared to non-stimulated (NS) WT $CysLT_2R$ analyzed by the Kruskal-Wallis nonparametric test followed by Dunn's correction). **(c)** LTD_4 stimulation significantly increases IP_1 production at the WT $CysLT_2R$ (***, $P < 0.001$ compared to non-stimulated (NS) $L129^{3.43}Q$ mutant, analyzed by the Kruskal-Wallis nonparametric test followed by Dunn's correction). In the absence of LTD_4 stimulation (NS), the $L129^{3.43}Q$ mutant produces IP_1 at levels resembling those of WT $CysLT_2R$ when activated by LTD_4. Application of LTD_4 to $L129^{3.43}Q$ does not further increase IP_1 production. The constitutive activity of $L129^{3.43}Q$ is not reversed following treatment with either cpd 11a or 11c. Data represent the mean ± s.d. of two independent experiments, performed in quadruplicate. Source data are provided as a Source Data file.

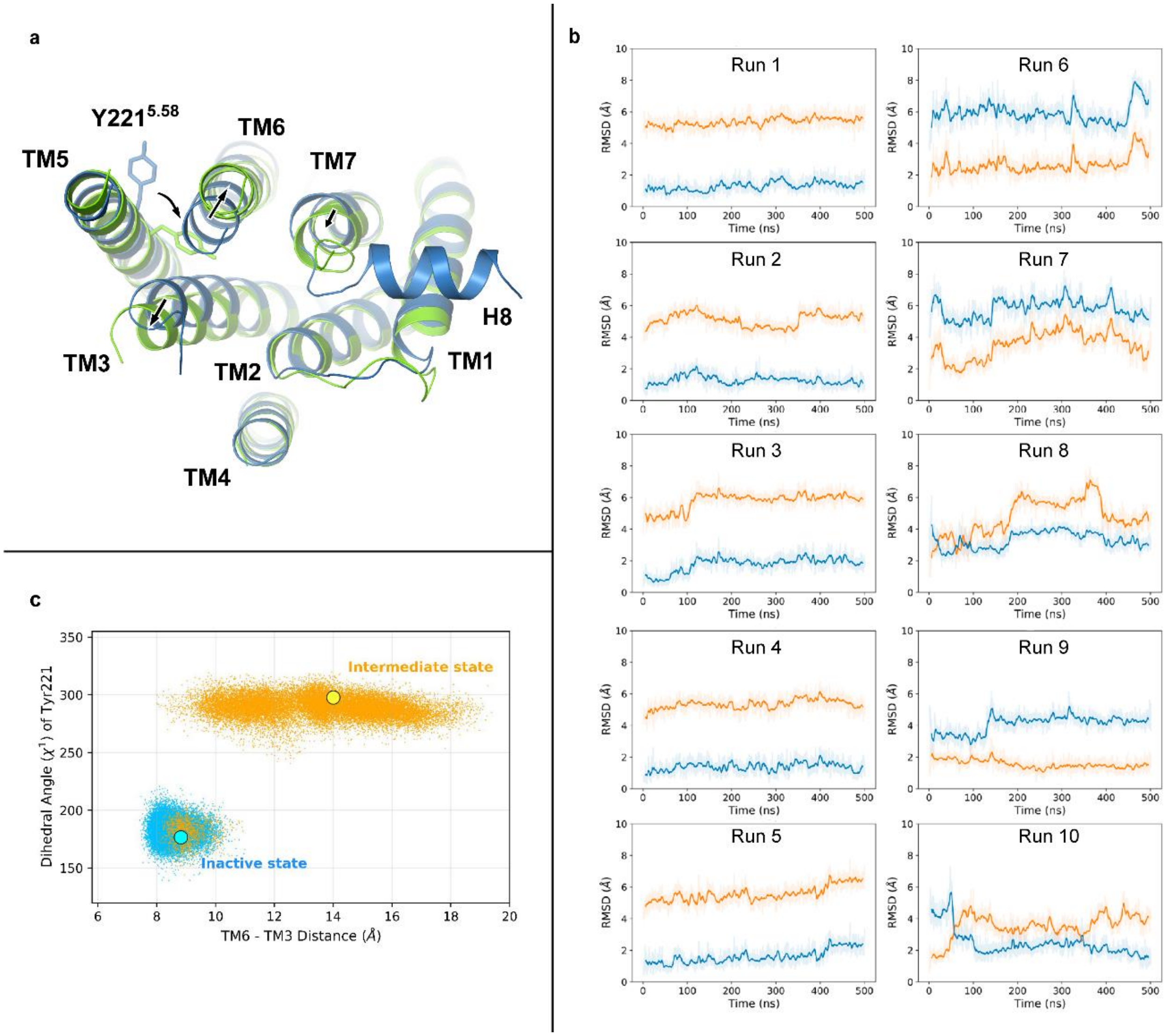

Supplementary Figure 7. Distinct conformation of CysLT$_2$R-11c. (a) Alignment between CysLT$_2$R-11a_C222$_1$ (blue) and CysLT$_2$R-11c (green) viewed from the intracellular side, demonstrating shifts of TM3, TM6, TM7, as well as the switch of the Y221$^{5.58}$ rotomer, consistent with transition into an intermediate active state. **(b)** MD simulations starting from the inactive and intermediate state structures of CysLT$_2$R. Root mean square deviation (RMSD) plots for TM6 backbone heavy atoms of residues Val240 – Ala245 compared to inactive (blue) and intermediate (orange) states. Both inactive state (runs 1 to 5) and intermediate state (runs 6 to 10) conformations were simulated for 500 ns each for a total of 10 independent runs providing a cumulative simulation time of 5 μs. Solid lines represent running average values obtained from 50 frames with sampling rate of 10 frames per ns. **(c)** Scatter plot of TM6 – TM3 distance vs. χ1 angle of Tyr221$^{5.58}$ as calculated from the MD data. TM distances were measured between centers of mass of Cα atoms of residues 241-244 (TM6) and 137-140 (TM3). The two small circles correspond to the inactive state (cyan) and the intermediate state (orange) structures. The plot shows the extent of conformational sampling of TM6 in the intermediate state which is distinct from a more confined conformational space explored in the inactive state.

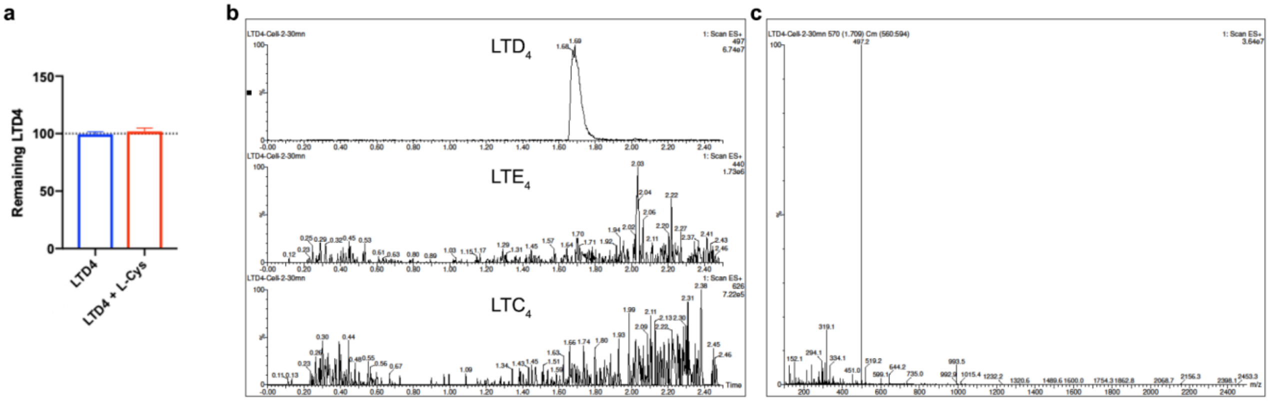

Supplementary Figure 8. No LTD$_4$ conversion was found in CysLT$_2$R functional assays. 10 µM of LTD$_4$ was incubated over a cell monolayer for 30 min with or without 10 mM of L-Cys (inhibitor of LTD$_4$ conversion) before quantification by UPLC/MS. (**a**) LTD$_4$ quantification after 30-min incubation at 37°C. Dotted line represent 100% as determined in stimulation buffer without cell incubation. No detectable conversion of LTD$_4$ was observed even in absence of L-Cys. Data represent mean ± s.e.m. of three independent experiments. (**b**) Mass extraction chromatogram for the masses of LTD$_4$, LTE$_4$, and LTC$_4$. While a sharp peak is observed for the mass of LTD$_4$, no above the background/noise peaks were observed for the masses of LTE$_4$ and LTC$_4$. (**c**) MS spectrum of the 1.70 min peak showing the M/Z of LTD$_4$ at 497.2 (reported MW is 496.7 Da).

Supplementary Table 1. Crystallographic data collection and refinement statistics.

Ligand<br>PDB ID	**Cpd 11a**<br>**6RZ6**	**Cpd 11a**<br>**6RZ7**	**Cpd 11b**<br>**6RZ9**	**Cpd 11c**<br>**6RZ8**
**Data collection**				
Number of crystals	49	10	4	7
Space group	$C222_1$	F222	$C222_1$	I4
Cell dimensions				
a, b, c (Å)	69.8, 170.9, 85.8	81.8, 142.1, 171.2	69.6, 170.2, 85.7	78.4, 78.4, 172.0
α, β, γ (°)	90.0, 90.0, 90.0	90.0, 90.0, 90.0	90.0, 90.0, 90.0	90.0, 90.0, 90.0
Resolution (Å)	30–2.43 (2.52–2.43)[a]	20–2.43 (2.52–2.43)	30–2.73 (2.83–2.73)	30–2.70 (2.80–2.70)
No. total reflection	180,906	67,591	38,974	199,739
No. unique reflections	19,685	17,665	13,531	14,227
R_{merge} (%)	27.9 (322.7)	19.4 (216.8)	15.1 (161.5)	23.9 (275.8)
R_{pim} (%)	9.4 (112.0)	10.8 (119.2)	10.2 (108.4)	6.6 (76.6)
Mean I/σ*I*	5.9 (1.1)	4.8 (0.7)	4.4 (0.9)	9.8 (1.2)
Completeness (%)	99.8 (99.7)	92.4 (89.2)	97.0 (99.1)	99.9 (100.0)
Multiplicity	9.2 (8.8)	3.8 (3.9)	2.9 (3.0)	14.0 (13.8)
CC* (%)	99.8 (67.8)	99.7 (59.7)	99.7 (70.8)	99.9 (76.3)
**Refinement**				
No. reflections/test set	19,661/982	17,555/883	13,514/674	14,226/709
R_{work}/R_{free} (%)	19.5/23.3 (28.3/30.7)	22.9/25.9 (33.2/33.2)	21.7/25.4 (32.4/35.8)	19.5/24.7 (28.1/32.9)
No. atoms				
Protein	2,798	2,740	2,740	2,764
Ligand	41	41	41	43
Lipids/other	487	351	325	196
Wilson *B*-factors (Å^2)	51.5	49.6	73.1	62.0
Mean overall *B* value (Å^2)				
$CysLT_2R$	57.5	55.0	74.9	69.9
BRIL	118.5	114.0	133.0	121.7
Ligand	49.4	47.8	82.5	61.2
Lipid and other	80.5	83.5	95.4	84.2
R.m.s. deviations				
Bond lengths (Å)	0.006	0.007	0.004	0.004
Bond angles (°)	1.04	1.11	0.81	0.72
Ramachandran stats (%)[b]				
Favored	98.58	97.73	98.86	97.50
Allowed	1.42	2.27	1.14	2.50
Outliers	0	0	0	0

[a]Values in parentheses are for highest-resolution shell.

[b]As defined by Molprobity[1]

Supplementary Table 2. Pairwise RMSD between $CysLT_1R$ and $CysLT_2R$ structures.

Structure		$CysLT_2R$				$CysLT_1R$	
		11a ($C222_1$)	11a (F222)	11b	11c	Pran	Zaf
$CysLT_2R$	11a ($C222_1$)	0	0.33	0.43	0.51	0.81	2.47
	11a (F222)	0.39	0	0.50	0.66	0.81	2.67
	11b	0.15	0.41	0	0.46	0.78	2.40
	11c	0.54	0.56	0.55	0	1.29	2.75
$CysLT_1R$	Pran	1.03	0.89	1.00	1.02	0	2.31
	Zaf	1.01	0.99	0.98	0.99	0.47	0

Bottom-left corner: Cα RMSD (in Å) on 90% of residues; top-right corner: RMSD (in Å) of all atoms with centers within 6 Å of the bound ligand, calculated after receptor alignment. Color intensity is proportional to the numerical value.

Supplementary Table 3. Structure-activity relationship (SAR) analysis.

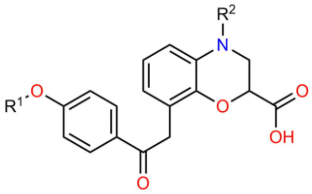

					Molecular Mechanism	
Cpd	R^1 (O – substitute)	R^2 (N – substitute)	IC_{50} (μM) CysLT$_1$R	IC_{50} (μM) CysLT$_2$R	Binding to CysLT$_1$R	Binding to CysLT$_2$R
**12**	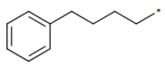	H	0.054	4.6	-	-
**13a**	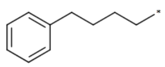	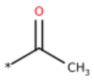	0.013	3.6	Elongation of the R_2 aliphatic chain results in potential clashes with $R22^{1.31}$, $Y26^{1.35}$, $V277^{7.35}$, and $Q274^{7.32}$ of the ligand binding pocket, and reduced binding affinity.	$Y26^{1.35}$ in CysLT$_1$R is replaced by smaller $F41^{1.35}$ in CysLT$_2$R. Also $L287^{7.35}$ side chain in CysLT$_2$R is shifted away relative to $V277^{7.35}$ of CysLT$_1$R. These changes expand CysLT$_2$R pocket to fit the elongated chain of the ligand.
**13b**	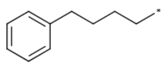	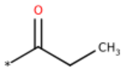	0.15	5.2		
**13c**	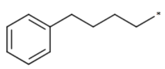	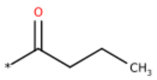	0.30	3.7		
**13d**	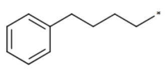	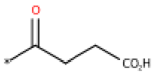	0.0014	0.89	In CysLT$_1$R, optimal interactions of the carboxylic chain $Y83^{2.64}$ and $Y26^{1.35}$ requires 3 carbons in the alkyl chain, like in 13e. Shorter (13d) or longer (13f) chain is suboptimal. Carbonyl oxygen may form a hydrogen bond with $H256^{6.58}$, which could explain higher affinity, as compared to 14a-c. Further elongation of the alkyl-carboxyl chain results in steric clashes (14b,c).	Carbonyl oxygen restricts the flexibility of the carboxyl group by fixing the bond angles, resulting in suboptimal interactions in CysLT$_2$R. Removing carbonyl group allows for carboxyl to form a new salt bridge to $K37^{1.31}$, dramatically improving binding to CysLT$_2$R (14a). Further elongation of the alkyl-carboxyl chain results in steric clashes (14b,c).
**13e**	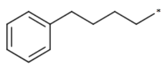	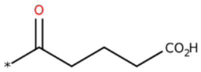	<0.0010	1.8		
**13f**	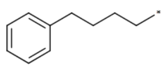	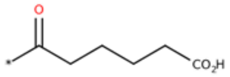	0.012	0.89		
**14a**	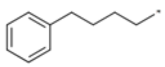	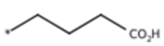	0.011	0.0025		
**14b**	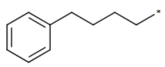	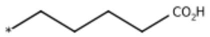	0.044	0.17		
**14c**	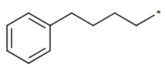	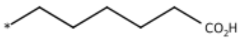	0.13	0.29		

**15a**	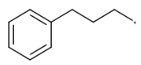	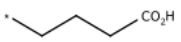	1.3	0.068	Addition of oxygen in R_1 introduces unfavorable polar-hydrophobic contact (compare 15a and 15c, also 14a vs 15d).	More open hydrophobic cleft in CysLT$_2$R can more easily accommodate the benzene ring, allowing for a longer length of the aliphatic chain.<br><br>For CysLT$_2$R the optimal length is 5 carbons, like in 15b.
**15b**	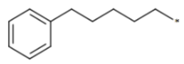	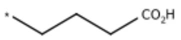	0.12	0.00062		
**15c**	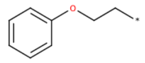	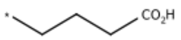	3.0	1.5		
**15d**	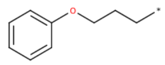	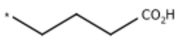	0.29	0.016		
**16**	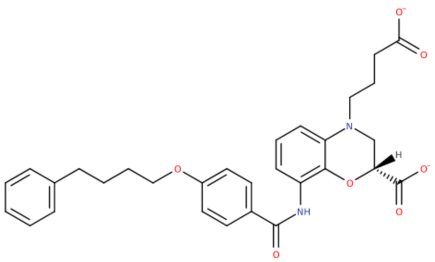		0.051	0.055	The number of hydrogen bonds does not depend on the stereoisomer.	Cpd 16 forms a single hydrogen bond to K194^{ECL2}, while stereo-isomer 17 allows for two hydrogen bonds with K194^{ECL2} and Y119$^{3.33}$.
**17**	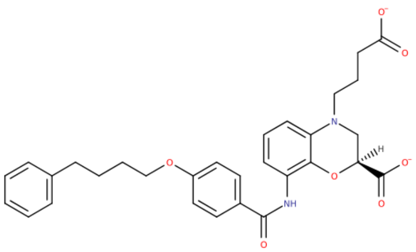		0.017	0.00087		
**18**	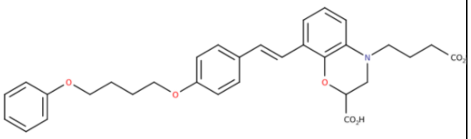		0.024	0.00031	Carbonyl oxygen in 19 flattens the ring system, while the oxy derivative 18 keeps the ring bent, which is more favorable due to steric constraints of the pocket.	Carbonyl oxygen in 19 flattens the ring system, while the oxy derivative 18 keeps the ring bent, which is more favorable due to steric constraints of the pocket.
**19**	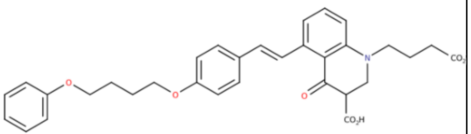		0.25	0.37		

SAR of O- and N- derivatives of the common 3,4-dihydro-2H-1,4-benzoxazine-2-carboxylic acid scaffold is based on their docking in CysLT$_1$R-pranlukast and CysLT$_2$R-11a structures. Cpds 12, 13a-f, 14a-c, 15a-d, 16, and 17 are cpds 1, 9a-f, 11a-c, 17a-d, 18, and 19 from ref.[2]; cpds 18 and 19 are cpds 12 and 13 from ref.[3]

Supplementary Table 4. Primers used in this study.

Primer name	Forward Primer (5'-to-3')	Reverse Primer (5'-to-3')
CysLT$_2$R crystallization construct		
M1-K322	CAAAAAGGTACCATGGAGAGAAAATTTATGTCCTG	CAAAAAAAGCTTTTTTCTGAGTGCAGACTTTAG
E232-BRIL-V240	CATTCGGGTTCTGTTAAAAGTGGAGGCTGATCTGGA AGACAATTGGGAAAC	GTCAGTGCCTTCCTGTGAGAAACCAGGTACTTCTGA ATGTATGCATTCCG
dHindIII	ACTGCATAAAGCCTTGGTTATCACACTGGCCTTG	GTGTGATAACCAAGGCTTTATGCAGTCTGTCTTTGC
M17	CCTGTATTTTCAGGGCGGTACCATGGAACCAAATG GCACCTTCAGC	GCTGAAGGTGCCATTTGGTTCCATGGTACCGCCCTG AAAATACAGG
W1.45V	CCCAATTGTATATCTGATAATATTTTTCGTTGGAGT CTTGGGAAATGGGTTGTC	GACAACCCATTTCCCAAGACTCCAACGAAAAATATT ATCAGATATACAATTGGG
F3.51Y	CTGACCGTGCTGAGTGTTGTGCGTTATCTGGCAATG GTTCACCCCTTTCGG	CCGAAAGGGGTGAACCATTGCCAGATAACGCACAA CACTCAGCACGGTCAG
D2.50N	CGTTTTCATGCTAAATCTGGCCATTTCAAACCTCCT GTTCATAAGCACGCTTCC	GGAAGCGTGCTTATGAACAGGAGGTTTGAAATGGCC AGATTTAGCATGAAAACG
CysLT$_2$R constructs for functional assays		
HAtag+M17	CCCATACGATGTTCCAGATTACGCTATGGAACCAA ATGGCACCTTCAGC	GCTGAAGGTGCCATTTGGTTCCATAGCGTAATCTGG AACATCGTATGGG
K322_pcDNA3.1	GGACAGACTAAAGTCTGCACTCAGAAAATAACTCG AGTCTAGAGGGCCCGTTTAAAC	GTTTAAACGGGCCCTCTAGACTCGAGTTATTTTCTGA GTGCAGACTTTAGTCTGTCC
K1.31R	CTGCACAATTGAAAACTTCCGTAGAGAATTTTTCCC AATTGTATATCTGATAATATTTTTCTGGGG	GATATACAATTGGGAAAAATTCTCTACGGAAGTTTT CAATTGTGCAGTTCCTGCTGTTGTTATTG
W1.45V	CCCAATTGTATATCTGATAATATTTTTCGTGGGAGT CTTGGGAAATGGGTTGTCCATATATG	CCCATTTCCCAAGACTCCCACGAAAAATATTATCAG ATATACAATTGGGAAAAATTCTCTCTTGAA
D2.50N	GCTAAATCTGGCCATTTCAAACCTCCTGTTCATAAG CACGCTTCCCTTCAG	CTGAAGGGAAGCGTGCTTATGAACAGGAGGTTTGAA ATGGCCAGATTTAGCATGAAAACGTTCAC
Y3.33F	GCAGGATTATGTCTTATTCCTTGTTTGTCAACATGT ACAGCAGTATTTATTTCCTGACC	GAAATAAATACTGCTGTACATGTTGACAAACAAGGA ATAAGACATAATCCTGCAGGCCAGG
I3.40V	GTCAACATGTACAGCAGTGTGTATTTCCTGACCGTG CTGAGTGTTGTG	CAGCACGGTCAGGAAATACACACTGCTGTACATGTT GACATACAAGG
Y3.41W	CAACATGTACAGCAGTATTTGGTTCCTGACCGTGCT GAGTGTTGTGCGTTTC	CTCAGCACGGTCAGGAACCAAATACTGCTGTACATG TTGACATACAAGG
L3.43Q	CAACATGTACAGCAGTATTTATTTCCAGACCGTGCT GAGTGTTGTGCGTTTCC	CAACACTCAGCACGGTCTGGAAATAAATACTGCTGT ACATGTTGACATACAAGGAATAAG
V3.49D	CCGTGCTGAGTGTTGACCGTTTCCTGGCAATGGTTC ACCCC	CCATTGCCAGGAAACGGTCAACACTCAGCACGGTCA GGAAATAAATAC
F3.51Y	GCTGAGTGTTGTGCGTTACCTGGCAATGGTTCACCC CTTTCGG	GGTGAACCATTGCCAGGTAACGCACAACACTCAGCA CGGTCAGG
S4.56A	GGATCCTTATCATGGCTGCTTCAATAATGCTCCTGG ACAGTGGCTCTGAG	CTGTCCAGGAGCATTATTGAAGCAGCCATGATAAGG ATCCATATGATCCCAC
K194N	CATGCTTAGAGCTGAATCTCTATAACATTGCTAAGC TGCAGACCATGAACTATATTG	GGTCTGCAGCTTAGCAATGTTATAGAGATTCAGCTC TAAGCATGATGTGACACTG
K194R	CATGCTTAGAGCTGAATCTCTATCTGATTGCTAAGC TGCAGACCATGAACTATATTG	GGTCTGCAGCTTAGCAATATAGAGATTCAGCTCTAA GCATGATGTGACACTG
L5.35A	GCTGAATCTCTATAAAATTGCTAAGGCGCAGACCA TGAACTATATTGCCTTGGTGGTGGGC	GGCAATATAGTTCATGGTCTGCGCCTTAGCAATTTTA TAGAGATTCAGCTCTAAGCATGATGTGAC
M5.38A	GCTAAGCTGCAGACCGCCAACTATATTGCCTTGGTG GTGGGCTGC	CCCACCACCAAGGCAATATAGTTGGCGGTCTGCAGC TTAGCAATTTTATAGAGATTCAG
M5.38L	TTGCTAAGCTGCAGACCCTGAACTATATTGCCTTGG TGGTGGGCTGC	CCACCAAGGCAATATAGTTCAGGGTCTGCAGCTTAG CAATTTTATAGAGATTCAG
M5.38V	GCTAAGCTGCAGACCGTGAACTATATTGCCTTGGTG GTGGG	CCAAGGCAATATAGTTCACGGTCTGCAGCTTAGCAA TTTTATAGAG
N5.39H	GCTAAGCTGCAGACCATGCATTATATTGCCTTGGTG GTGGGCTGCCTGC	CCACCACCAAGGCAATATAATGCATGGTCTGCAGCT TAGCAATTTTATAGAGATTCAGCTC
F6.48W	GATCATCTTCTTCTTGTGTTGGCTGCCCTATCACAC ACTGAGGACC	GTGTGTGATAGGGCAGCCAACACAAGAAGAAGATG ATCAAGGTGATGATG
R6.55K	GCCCTATCACACACTGAAAACCGTCCACTTGACGA CATGGAAAGTGGG	CGTCAAGTGGACGGTTTTCAGTGTGTGATAGGGCAG GAAACACAAGAAGAAG
H7.32Q	GGTTTATGCAAAGACAGACTGCAGAAAGCTTTGGT TATCACACTGGCCTTGGCAGCAGCC	CAGTGTGATAACCAAAGCTTTCTGCAGTCTGTCTTTG CATAAACCCACTTTCCATGTCG
N7.45C	CTTGGCAGCAGCCTGCGCCTGCTTCAATCCTCTGCT CTATTAC	GAGGATTGAAGCAGGCGCAGGCTGCTGCCAAGGCC AGTGTG
N7.49D	CCAATGCCTGCTTCGACCCTCTGCTCTATTACTTTGC TGGGG	CAAAGTAATAGAGCAGAGGGTCGAAGCAGGCATTG GCTGCTGCCAAG
E8.48A	GCTCTATTACTTTGCTGGGGCTAATTTTAAGGACAG ACTAAAGTCTGCACTCAG	GACTTTAGTCTGTCCTTAAAATTACCCCCAGCAAAG TAATAGAGCAGAGGATTGAAG
E8.48G	GCTCTATTACTTTGCTGGGGGTAATTTTAAGGACAG ACTAAAGTCTGCACTCAG	GACTTTAGTCTGTCCTTAAAATTAGCCCCAGCAAAG TAATAGAGCAGAGGATTGAAG

Supplementary Table 5. DNA sequence of the $CysLT_2R$ crystallization construct.

ATGAAGACGATCATCGCCCTGAGCTACATCTTCTGCCTGGTATTCGCCGACTACAAGGACGATGATGACGCCAAACTGCAGACCATGCATCATCATCATCATCATCATCATCATCATGAAAACCTGTATTTTCAGGGCGGTACCatggaaccaaatggcaccttcagcaataacaacagcaggaactgcacaattgaaaacttcaagagagaatttttcccaattgtatatctgataatatttttcgttggagtcttgggaaatgggttgtccatatatgttttcctgcagccttataagaagtccacatctgtgaacgttttcatgctaaatctggccatttcaaacctcctgttcataagcacgcttcccttcagggctgactattatcttagaggctccaattggatatttggagacctggcctgcaggattatgtcttattccttgtatgtcaacatgtacagcagtatttatttcctgaccgtgctgagtgttgtgcgttatctggcaatggttcacccctttcggcttctgcatgtcaccagcatcaggagtgcctggatcctctgtgggatcatatggatccttatcatggcttcctcaataatgctcctggacagtggctctgagcagaacggcagtgtcacatcatgcttagagctgaatctctataaaattgctaagctgcagaccatgaactatattgccttggtggtgggctgcctgctgccatttttcacactcagcatctgttatctgctgatcattcgggttctgttaaaagtggaggctgatctggaagacaattgggaaactctgaacgacaatctcaaggtgatcgagaaggctgacaatgctgcacaagtcaaagacgctctgaccaagatgagggcagcagccctggacgctcagaaggccactccacctaagctcgaggacaagagcccagatagccctgaaatgaaagactttcggcatggattcgacattctggtgggacagattgatgatgcactcaagctggccaatgaagggaaagtcaaggaagcacaagcagccgctgagcagctgaagaccacccggaatgcatacattcagaagtacctggtttctcacaggaaggcactgaccaccatcatcatcaccttgatcatcttcttcttgtgtttcctgccctatcacacactgaggaccgtccacttgacgacatggaaagtgggtttatgcaaagacagactgcataaagccttggttatcacactggccttggcagcagccaatgcctgcttcaatcctctgctctattactttgctggggagaattttaaggacagactaaagtctgcactcagaaaaTAA

Sequences of the cleaved tags and the stop codon are shown in capital letters.

Supplementary Methods

Ligand synthesis and characterization

The overall ligand synthesis scheme is shown in Supplementary Fig. 1. Analytical samples were homogeneous, as confirmed by TLC, and afforded spectroscopic results consistent with the assigned structures. Proton and carbon nuclear magnetic resonance spectra (^1H and ^{13}C NMR) were taken on a Varian Mercury 300 spectrometer using deuterated chloroform ($CDCl_3$) and deuterated dimethylsulfoxide (DMSO-d_6) as the solvent. Fast atom bombardment (FAB) mass spectra were obtained on a JEOL JMS-DX303HF spectrometer. Electrospray ionization (ESI, HRMS) mass spectra were obtained on a Thermo Fisher Scientific LTQ Orbitrap XL system. Column chromatography was carried out on silica gel (Merck Silica Gel 60, Wako gel C-200, or Fuji Silysia FL60D). Thin layer chromatography was performed on silica gel (Merck TLC or HPTLC plates, Silica Gel 60 F254). The following abbreviations for solvents and reagents are used: *N*,*N*-dimethylformamide (DMF), ethanol (EtOH), ethyl acetate (EtOAc), methanol (MeOH), tetrahydrofuran (THF), and dimethoxyethane (DME).

benzyl 2-hydroxy-3-[[(2*R*)-2-hydroxy-3-methoxy-3-oxopropyl]amino]benzoate (3)

A solution of benzyl 3-amino-2-hydroxybenzoate **1** (7.35 g, 30.2 mmol) and methyl (2*R*)-glycidate **2** (3.08 g, 30.2 mmol) in EtOH (30 mL) was stirred under reflux for 4 h. After cooling the reaction solution to room temperature (RT), the solvent was evaporated and the residual dark brown oil was treated with EtOH (25 mL) and hexane (25 mL). The precipitates were collected by filtration to afford **3** (4.35 g, 42%) as a white powder.

^1H NMR (300 MHz, $CDCl_3$) δ 3.04 (d, J = 5.1 Hz, 1H), 3.44-3.53 (m, 1H), 3.53-3.64 (m, 1H), 3.80 (s, 3H), 4.40-4.48 (m, 1H), 4.72 (t, J = 6.6 Hz, 1H), 5.37 (s, 2H), 6.77 (t, J = 7.9 Hz, 1H), 6.81-6.87 (m, 1H), 7.25 (dd, J = 7.9, 1.6 Hz, 1H), 7.32-7.49 (m, 5H), 11.03 (s, 1H).

8-*O*-benzyl 2-*O*-methyl (2*S*)-3,4-dihydro-2*H*-1,4-benzoxazine-2,8-dicarboxylate (4)

To a solution of **3** (4.92 g, 14.2 mmol) in THF (25 mL) was successively added diethyl azodicarboxylate (6.4 mL, 17.3 mmol) and a solution of triphenylphosphine (4.53 g, 17.3 mmol) in THF (5 mL) at 0 °C. The reaction mixture was stirred for 30 min at 0 °C and for 30 min at RT. Then the reaction mixture was treated with triphenylphosphine (1.12 g, 4.3 mmol) and diethyl azodicarboxylate (1.6 mL, 3.1 mmol). The resulting mixture was poured into water and extracted with EtOAc. The organic layer was washed sequentially with water and brine, dried over anhydrous sodium sulfate, and concentrated. The residue was purified by column chromatography on silica gel (n-hexane/EtOAc) to give **4** (3.92 g, 84%).

^1H NMR (300 MHz, $CDCl_3$) δ 3.74-3.76 (m, 3H), 3.88 (s, 3H), 4.99 (t, J = 3.3 Hz, 1H), 5.32 (d, J = 12.6 Hz, 1H), 5.37 (d, J = 12.6 Hz, 1H), 6.72 (dd, J = 7.9, 1.8 Hz, 1H), 6.75-6.85 (m, 1H), 7.27-7.40 (m, 4H), 7.42-7.50 (m, 2H).

8-*O*-benzyl 2-*O*-methyl (2*S*)-4-(4-ethoxy-4-oxobutyl)-2,3-dihydro-1,4-benzoxazine-2,8-dicarboxylate (5)

To a solution of **4** (3.89 g, 11.9 mmol) and ethyl 4-oxobutanoate (2.63 g, 20.2 mmol) in THF (34 mL) and acetic acid (8.5 mL) was added $NaBH(OAc)_3$ (6.28 g, 29.7 mmol) at 0 °C, and the mixture was stirred for 3.5 h at RT. After further addition of ethyl 4-oxobutanoate (658 mg, 5.1 mmol) and $NaBH(OAc)_3$ (1.57 g, 7.4 mmol), the mixture was stirred for 1.5 h at RT. The reaction mixture was poured into saturated aqueous NH_4Cl, and the resulting mixture was extracted with EtOAc twice. The combined organic layer was washed sequentially with water, saturated aqueous sodium bicarbonate

and brine, dried over anhydrous sodium sulfate, and concentrated. The residue was purified by column chromatography on silica gel (n-hexane/EtOAc) to give **5** (3.81 g, 73%).

^{1}H NMR (300 MHz, $CDCl_3$) δ 1.26 (t, *J* = 7.1 Hz, 3H), 1.83-1.97 (m, 2H), 2.34 (t, *J* = 7.0 Hz, 2H), 3.26-3.33 (m, 2H), 3.56 (d, *J* = 3.7 Hz, 2H), 3.74 (s, 3H), 4.14 (q, *J* = 7.1 Hz, 2H), 4.97 (t, *J* = 3.8 Hz, 1H), 5.32 (d, *J* = 12.6 Hz, 1H), 5.37 (d, *J* = 12.6 Hz, 1H), 6.80-6.90 (m, 2H), 7.16-7.24 (m, 1H), 7.28-7.41 (m, 3H), 7.42-7.52 (m, 2H).

(2*S*)-4-(4-ethoxy-4-oxobutyl)-2-methoxycarbonyl-2,3-dihydro-1,4-benzoxazine-8-carboxylic acid (6)

A mixture of **5** (3.80 g, 8.61 mmol), 10% palladium-carbon (380 mg) and EtOH (29 mL) was stirred for 3 h under hydrogen atmosphere. The catalyst was filtered off, and the filtrate was concentrated to give **6** (2.98 g, 99%) as crude orange oil.

^{1}H NMR (300 MHz, $CDCl_3$) δ 1.26 (t, *J* = 7.1 Hz, 3H), 1.85-2.00 (m, 2H), 2.33-2.41 (m, 2H), 3.34 (dd, *J* = 8.1, 6.9 Hz, 2H), 3.58 (dd, *J* = 12.3, 5.5 Hz, 1H), 3.69 (dd, *J* = 12.3, 3.3 Hz, 1H), 3.83 (s, 3H), 4.15 (q, *J* = 7.1 Hz, 2H), 5.03 (dd, *J* = 5.4, 3.4 Hz, 1H), 6.93 (dd, *J* = 8.2, 1.8 Hz, 1H), 6.96-7.04 (m, 1H), 7.50 (dd, *J* = 7.5, 1.8 Hz, 1H).

methyl (2*S*)-4-(4-ethoxy-4-oxobutyl)-8-(phenylmethoxycarbonylamino)-2,3-dihydro-1,4-benzoxazine-2-carboxylate (7)

A solution of **6** (2.98 g, 8.48 mmol), diphenylphosphoryl azide (2.45 g, 8.90 mmol), benzyl alcohol (1.01 g, 9.35 mmol), and triethylamine (1.24 mL, 8.90 mmol) in toluene (28 mL) was stirred for 30 min at 60 °C and for 4 h at 80 °C. The mixture was cooled to RT, poured into water, and extracted with EtOAc twice. The combined organic layer was washed with brine, dried over anhydrous sodium sulfate, and concentrated. The residue was purified by column chromatography on silica gel (n-hexane/EtOAc) to give **7** (2.37 g, 61%).

^{1}H NMR (300 MHz, $CDCl_3$) δ 1.25 (t, *J* = 7.3 Hz, 3H), 1.82-1.96 (m, 2H), 2.34 (t, *J* = 7.1 Hz, 2H), 3.17-3.37 (m, 2H), 3.50 (d, *J* = 4.0 Hz, 2H), 3.75-3.77 (m, 3H), 4.13 (q, *J* = 7.1 Hz, 2H), 4.82 (t, *J* = 4.0 Hz, 1H), 5.13-5.27 (m, 2H), 6.43 (dd, *J* = 8.4, 1.3 Hz, 1H), 6.83 (t, *J* = 8.3 Hz, 1H), 7.29-7.46 (m, 6H), 7.53 (d, *J* = 7.7 Hz, 1H).

methyl (2*S*)-8-amino-4-(4-ethoxy-4-oxobutyl)-2,3-dihydro-1,4-benzoxazine-2-carboxylate (8)

A mixture of **7** (2.35 g, 5.15 mmol), 10% palladium-carbon (470 mg) in EtOH (17 mL) was stirred for 1 h at 40 °C under atmosphere of hydrogen. The catalyst was filtered off, and the filtrate was concentrated. The residue was purified by column chromatography on silica gel (*n*-hexane/EtOAc) to give **8** (1.30 g, 78%).

^{1}H NMR (300 MHz, $CDCl_3$) δ 1.25 (t, *J* = 7.1 Hz, 3H), 1.82-1.97 (m, 2H), 2.34 (t, *J* = 7.1 Hz, 2H), 3.14-3.37 (m, 2H), 3.42-3.56 (m, 2H), 3.76 (brs, 2H), 3.77 (s, 3H), 4.13 (q, *J* = 7.1 Hz, 2H), 4.82 (dd, *J* = 4.8, 3.6 Hz, 1H), 6.12-6.23 (m, 2H), 6.66 (t, *J* = 8.1 Hz, 1H).

methyl (2*S*)-8-[[4-[4-(2,3-difluorophenoxy)butoxy]-2-fluorobenzoyl]amino]-4-(4-ethoxy-4-oxobutyl)-2,3-dihydro-1,4-benzoxazine-2-carboxylate (10c)

A mixture of **8** (100 mg, 0.30 mmol), 4-[4-(2,3-difluorophenoxy)butoxy]-2-fluorobenzoic acid **9c** (122 mg, 0.36 mmol), 1-(3-Dimethylaminopropyl)-3-ethylcarbodiimide hydrochloride (69 mg, 0.36 mmol), 1-hydroxybenzotriazole (49 mg, 0.36 mmol), and N,N-dimethyl-4-aminopyridine (37 mg, 0.30 mmol) in DMF (1 mL) was stirred overnight at RT. The reaction mixture was poured into water and extracted with EtOAc. The organic layer was washed sequentially with water and saturated brine,

dried over anhydrous sodium sulfate, and concentrated. The residue was purified by column chromatography on silica gel (*n*-hexane/EtOAc) to give **10c** (148 mg, 77%).

^{1}H NMR (300 MHz, $CDCl_3$) δ 1.25 (t, *J* = 7.2 Hz, 3H), 1.88-1.97 (m, 2H), 2.03-2.11 (m, 4H), 2.36 (t, *J* = 7.2 Hz, 2H), 3.27-3.35 (m, 2H), 3.49-3.61 (m, 2H), 3.81 (s, 3H), 4.10-4.18 (m, 6H), 4.89-4.92 (m, 1H), 6.50-6.52 (m, 1H), 6.64-7.00 (m, 6H), 7.97 (d, *J* = 8.1 Hz, 1H), 8.13 (t, *J* = 9.0 Hz, 1H), 9.17 (d, *J* = 16.0 Hz, 1H).

(2*S*)-4-(3-carboxypropyl)-8-[[4-[4-(2,3-difluorophenoxy)butoxy]-2-fluorobenzoyl]amino]-2,3-dihydro-1,4-benzoxazine-2-carboxylic acid (11c = ONO-2080365)

To a solution of **10c** (151 mg, 0.23 mmol) in DME (2.4 mL) was added 2 mol/L aqueous solution of sodium hydroxide (0.53 mL), and the mixture was stirred for 5 h at RT. The reaction mixture was acidified with 1 mol/L hydrochloric acid and extracted with EtOAc. The organic layer was washed sequentially with water twice and brine, dried over anhydrous magnesium sulfate, and concentrated. The residue was recrystallized from EtOAc and hexane to give **11c** (125 mg, 89%)

^{1}H NMR (300 MHz, DMSO-d_6) δ 1.65 - 1.78 (m, 2 H), 1.83 - 1.96 (m, 4 H), 2.27 (t, *J* = 7.2 Hz, 2 H), 3.19 - 3.29 (m, 2 H), 3.48 (d, *J* = 3.7 Hz, 2 H), 4.07 - 4.23 (m, 4 H), 5.05 (t, *J* = 3.2 Hz, 1 H), 6.55 (dd, J=8.3, 1.2 Hz, 1 H), 6.76 (t, *J* = 8.1 Hz, 1 H), 6.89 - 7.20 (m, 5 H), 7.59 (d, *J* = 8.2 Hz, 1 H), 7.89 (t, *J* = 9.1 Hz, 1 H), 9.12 (d, *J* = 12.8 Hz, 1 H), 12.13 (s, 1 H), 13.04 (s, 1 H). ^{13}C NMR (150 MHz, DMSO-d_6) δ 174.3, 170.2, 162.7 (d, *J* = 13 Hz), 160.8 (d, *J* = 246 Hz), 160.3, 150.5 (dd, *J* = 244, 10 Hz), 148.1 (dd, *J* = 8, 3 Hz), 140.2 (dd, *J* = 245, 14 Hz), 134.4, 132.4 (d, *J* = 3 Hz), 131.8, 126.6, 124.1 (dd, *J* = 9, 5 Hz), 120.8, 113.7 (d, *J* = 11 Hz), 111.7, 110.5 (d, *J* = 2 Hz), 109.4, 108.8 (d, *J* = 17 Hz), 107.7, 102.3 (d, *J* = 28 Hz), 71.8, 68.8, 68.1, 49.1, 47.2, 30.7, 25.1, 25.0, 20.9. MS (FAB, Pos.) *m/z* 603 $(M + H)^+$. HRMS (ESI) $C_{30}H_{30}O_8N_2F_3$ $(M + H)^+$ calc. mass 603.1949, found 603.1949.

methyl (2*S*)-8-[[4-[4-(2-chloro-5-fluorophenyl)butoxy]-2-fluorobenzoyl]amino]-4-(4-ethoxy-4-oxobutyl)-2,3-dihydro-1,4-benzoxazine-2-carboxylate (10a)

10a (220 mg, 75%) was prepared under the same procedure as **10c** by using **9a** instead of **9c**.

^{1}H NMR (300 MHz, $CDCl_3$) δ 1.25 (t, *J* = 7.5 Hz, 3H), 1.80-2.00 (m, 6H), 2.38 (t, *J* = 7.5 Hz, 2H), 2.78-2.82 (m, 2H), 3.20-3.40 (m, 2H), 3.50-3.60 (m, 2H), 3.80 (s, 3H), 4.05 (t, *J* = 7.5 Hz, 2H), 4.18 (q, *J* = 7.5 Hz, 2H), 4.95 (dd, *J* = 3.0, 1.0 Hz, 1H), 6.50 (dd, *J* = 5.0, 1.0 Hz, 1H), 6.80-7.00 (m, 5H), 7.20-7.35 (m, 1H), 7.80-8.00 (m, 3H), 8.50 (brs, 1H).

(2*S*)-4-(3-carboxypropyl)-8-[[4-[4-(2-chloro-5-fluorophenyl)butoxy]benzoyl]amino]-2,3-dihydro-1,4-benzoxazine-2-carboxylic acid (11a = ONO-2570366)

11a (170 mg, 83%) was prepared under the same procedure as **11c**

^{1}H NMR (300 MHz, DMSO-d_6) δ 1.63 - 1.88 (m, 6 H), 2.27 (t, *J* = 7.1 Hz, 2 H), 2.76 (t, *J* = 7.1 Hz, 2 H), 3.16 - 3.60 (m, 4 H), 4.08 (t, *J* = 5.7 Hz, 2 H), 5.01 (t, *J* = 3.2 Hz, 1 H), 6.59 (d, *J* = 7.9 Hz, 1 H), 6.77 (t, *J* = 7.9 Hz, 1 H), 6.98 - 7.13 (m, 3 H), 7.17 (d, *J* = 7.9 Hz, 1 H), 7.27 (dd, *J* = 9.6, 3.2 Hz, 1 H), 7.45 (dd, *J* = 9.0, 5.3 Hz, 1 H), 7.88 (d, *J* = 8.8 Hz, 2 H), 9.21 (s, 1 H), 11.53 - 13.32 (m, 2 H). MS (FAB, Pos.) *m/z* 585 $(M + H)^+$. ^{13}C NMR (150 MHz, DMSO-d_6) δ 174.3, 170.4, 164.2, 161.4, 160.8 (d, *J* = 244 Hz), 141.8 (d, *J* = 7 Hz), 134.7, 133.9, 130.8 (d, *J* = 8 Hz), 129.2, 128.0, 126.4, 126.3, 120.6, 117.3 (d, *J* = 23 Hz), 114.7 (d, *J* = 22 Hz), 114.3, 111.9, 108.3, 71.9, 67.4, 49.2, 47.1, 32.4, 30.7, 28.1, 25.5, 20.9. HRMS (ESI) $C_{30}H_{30}O_8N_2F_3$ $(M + H)^+$ calc. mass 585.1798, found 585.1798.

methyl (2*S*)-4-(4-ethoxy-4-oxobutyl)-8-[[2-fluoro-4-[4-(5-fluoro-2-methylphenyl)butoxy]benzoyl]amino]-2,3-dihydro-1,4-benzoxazine-2-carboxylate (10b)

10b (150 mg, 60%) was prepared under the same procedure as **10c** by using **9b** instead of **9c**.

^{1}H NMR (300 MHz, $CDCl_3$) δ 1.22 (t, *J* = 7.1 Hz, 3H), 1.70-2.00 (m, 6H), 2.18 (t, *J* = 7.1 Hz, 2H), 2.22 (s, 3H), 2.65 (t, *J* = 7.2 Hz, 2H), 3.20-3.40 (m, 2H), 3.50-3.60 (m, 2H), 3.80 (s, 3H), 4.02-4.09 (m, 2H), 4.09-4.18 (m, 2H), 4.90 (dd, *J* = 5.0, 3.4 Hz, 1H), 6.45 (dd, *J* = 8.2, 1.3 Hz, 1H), 6.75-6.95 (m, 5H), 7.07 (dd, *J* = 8.2, 6.4 Hz, 1H), 7.80-7.95 (m, 3H), 8.45 (brs, 1H).

(2*S*)-4-(3-carboxypropyl)-8-[[4-[4-(5-fluoro-2-methylphenyl)butoxy]benzoyl]amino]-2,3-dihydro-1,4-benzoxazine-2-carboxylic acid (11b = ONO-2770372)

11b (81 mg, 57%) was prepared under the same procedure as **11c**

^{1}H NMR (300 MHz, DMSO-d_6) δ 1.88-2.16 (m, 6 H), 2.38-2.68 (m, 2 H), 2.76 (s, 3 H), 2.83-2.96 (m, 2 H), 3.43 - 3.83 (m, 4 H), 4.26-4.42 (m, 2 H), 5.22-5.32 (m, 1 H), 6.85 (d, *J* = 8.3 Hz, 1 H), 7.03 (t, *J* = 8.3 Hz, 1 H), 7.09-7.57 (m, 6 H), 8.14 (d, *J* = 8.1 Hz, 2 H), 9.46 (s, 1 H), 12.31-13.19 (m, 2 H). MS (FAB, Pos.) *m/z* 565 $(M + H)^+$. ^{13}C NMR (150 MHz, DMSO-d_6) δ 174.3, 170.4, 164.2, 161.4, 160.6 (d, *J* = 242 Hz), 142.7 (d, *J* = 7 Hz), 134.7, 133.9, 131.5 (d, *J* = 2 Hz), 131.3 (d, *J* = 8 Hz), 129.2, 126.4, 126.3, 120.6, 115.0 (d, *J* = 21 Hz), 114.3, 112.2 (d, *J* = 20 Hz), 111.9, 108.3, 71.9, 67.5, 49.2, 47.2, 32.0, 30.7, 28.3, 25.7, 20.9, 18.1. HRMS (ESI) $C_{30}H_{30}O_8N_2F_3$ $(M + H)^+$ calc. mass 565.2345, found 565.2344.

Supplementary References

1. Chen, V. B. *et al.* MolProbity : all-atom structure validation for macromolecular crystallography. *Acta Crystallogr. Sect. D Biol. Crystallogr.* **66**, 12–21 (2010).
2. Itadani, S. *et al.* Discovery of highly potent dual CysLT1 and CysLT2 antagonist. *ACS Med. Chem. Lett.* **5**, 1230–1234 (2014).
3. Itadani, S. *et al.* Discovery of a potent, orally available dual CysLT1 and CysLT2 antagonist with dicarboxylic acid. *Bioorganic Med. Chem.* **23**, 2079–2097 (2015).
