# Supplementary material for: Structural basis of ligand selectivity and disease mutations in cysteinyl leukotriene receptors
Source: Nat Commun. 2019 Dec 6;10:5573. doi: 10.1038/s41467-019-13348-2 (PMC6897958; doi:10.1038/s41467-019-13348-2)
Supplement: Supplementary file 3 — Reporting Summary [file 41467_2019_13348_MOESM3_ESM.pdf]

nature research

Corresponding author(s): Cherezov V, Mishin A, Sarret P

Last updated by author(s): Oct 28, 2019

Reporting Summary

Nature Research wishes to improve the reproducibility of the work that we publish. This form provides structure for consistency and transparency in reporting. For further information on Nature Research policies, see Authors & Referees and the Editorial Policy Checklist .

Statistics

For all statistical analyses, confirm that the following items are present in the figure legend, table legend, main text, or Methods section.

n/a	Confirmed	
☐	☒	The exact sample size (*n*) for each experimental group/condition, given as a discrete number and unit of measurement
☐	☒	A statement on whether measurements were taken from distinct samples or whether the same sample was measured repeatedly
☐	☒	The statistical test(s) used AND whether they are one- or two-sided<br>*Only common tests should be described solely by name; describe more complex techniques in the Methods section.*
☒	☐	A description of all covariates tested
☒	☐	A description of any assumptions or corrections, such as tests of normality and adjustment for multiple comparisons
☐	☒	A full description of the statistical parameters including central tendency (e.g. means) or other basic estimates (e.g. regression coefficient) AND variation (e.g. standard deviation) or associated estimates of uncertainty (e.g. confidence intervals)
☒	☐	For null hypothesis testing, the test statistic (e.g. *F*, *t*, *r*) with confidence intervals, effect sizes, degrees of freedom and *P* value noted<br>*Give P values as exact values whenever suitable.*
☒	☐	For Bayesian analysis, information on the choice of priors and Markov chain Monte Carlo settings
☒	☐	For hierarchical and complex designs, identification of the appropriate level for tests and full reporting of outcomes
☒	☐	Estimates of effect sizes (e.g. Cohen's *d*, Pearson's *r*), indicating how they were calculated

Our web collection on statistics for biologists contains articles on many of the points above.

Software and code

Policy information about availability of computer code

Data collection	Crystallographic data at ESRF were collected using MXCuBe software
Data analysis	NCBI blastp server (https://blast.ncbi.nlm.nih.gov/Blast.cgi?PAGE=Proteins; queried in 2015-2018)<br>Pubchem server (https://pubchem.ncbi.nlm.nih.gov/, queried in 2017-2018)<br>Prodrg server (http://davapc1.bioch.dundee.ac.uk/cgi-bin/prodrg/submit.html; queried in 2017-2018)<br>CHARMM-GUI web-server (http://www.charmm-gui.org/; queried in January 2018)<br>GPCRdb (http://gpcrdb.org/; queried in 2015-2019)<br>MolProbity server v.4.4 (http://molprobity.biochem.duke.edu/; queried in 2018)<br>QC Check server v.3.1 (https://smb.slac.stanford.edu/jcsg/QC/; queried in 2018)<br>OPM database (http://opm.phar.umich.edu; queried in January 2018)<br>UniProt (https://www.uniprot.org/; queried in 2018)<br>Grade web server (http://grade.globalphasing.org/cgi-bin/grade/server.cgi; queried in 2017-2018)<br>BEST v.3.1; XDS v.June1 2017; Phaser v.2.1; Buster v.2.10.2; PHENIX-1.9.1692; WinCoot v.0.8.6; CCP4 v.7.0.044; Rotor-Gene Q v.2.3.1.49; GraphPad Prism v.7.0; ICM-Pro v.3.8-6; PyMOL v.1.3; Python v.2.7; GROMACS v.2018.1

For manuscripts utilizing custom algorithms or software that are central to the research but not yet described in published literature, software must be made available to editors/reviewers. We strongly encourage code deposition in a community repository (e.g. GitHub). See the Nature Research guidelines for submitting code & software for further information.

Data

Policy information about availability of data

All manuscripts must include a data availability statement. This statement should provide the following information, where applicable:

- Accession codes, unique identifiers, or web links for publicly available datasets
- A list of figures that have associated raw data
- A description of any restrictions on data availability

The source data underlying Supplementary Figs. 5 and 6 are provided as a Source Data file. Other Data supporting the findings of this manuscript are available from the corresponding authors upon reasonable request. A reporting summary for this Article is available as a Supplementary Information file.
Coordinates and structure factors have been deposited in the Protein Data Bank (PDB) under the accession codes 6RZ6 (CysLT2R-11a, C2221 space group), 6RZ7 (CysLT2R-11a, F222 space group), 6RZ8 (CysLT2R-11c), 6RZ9 (CysLT2R-11b).

Field-specific reporting

Please select the one below that is the best fit for your research. If you are not sure, read the appropriate sections before making your selection.

[x] Life sciences   [ ] Behavioural & social sciences   [ ] Ecological, evolutionary & environmental sciences

For a reference copy of the document with all sections, see nature.com/documents/nr-reporting-summary-flat.pdf

Life sciences study design

All studies must disclose on these points even when the disclosure is negative.

Sample size	No statistical methods were used to predetermine sample size.
Data exclusions	No data were excluded.
Replication	For functional data, the number of independent experiments performed in quadruplicate is shown in Table 1. All attempts at replication were succesfull and presented.
Randomization	This study did not allocate samples in experimental groups thus no randomization was required for the reported experiments.
Blinding	The researchers were not blinded to allocation during experiments and outcome assessment. Blinding was not required for the reported experiments because all functional and structural data were analyzed using the same methods, and results are not subjective.

Reporting for specific materials, systems and methods

We require information from authors about some types of materials, experimental systems and methods used in many studies. Here, indicate whether each material, system or method listed is relevant to your study. If you are not sure if a list item applies to your research, read the appropriate section before selecting a response.

Materials & experimental systems

n/a	Involved in the study	
[ ]	[x]	Antibodies
[ ]	[x]	Eukaryotic cell lines
[x]	[ ]	Palaeontology
[x]	[ ]	Animals and other organisms
[x]	[ ]	Human research participants
[x]	[ ]	Clinical data

Methods

n/a	Involved in the study	
[x]	[ ]	ChIP-seq
[x]	[ ]	Flow cytometry
[x]	[ ]	MRI-based neuroimaging

Antibodies

Antibodies used	mouse monoclonal anti-HA tag antibody coupled to HRP (clone 12CA5, Roche); ANTI-FLAG M2-FITC (Sigma)
Validation	Validated by manufacturer.

Eukaryotic cell lines

Policy information about cell lines

Cell line source(s)	Sf9 cells were purchased from MilliporeSigma (Novagen, cat 71104), HEK293 cells were were purchased from the American Type Culture Collection (ATCC CRL-1573).
Authentication	The cell lines were authenticated by the suppliers.
Mycoplasma contamination	Both Sf9 and HEK293 cells have been tested and shown to be free from mycoplasma.
Commonly misidentified lines (See ICLAC register)	No commonly misidentified cell lines were used.
